# Oncogenic *GNAQ* and *GNA11* Mutations in Uveal Melanoma in Chinese

**DOI:** 10.1371/journal.pone.0109699

**Published:** 2014-10-03

**Authors:** Xiaolin Xu, Wen Bin Wei, Bin Li, Fei Gao, Zhibao Zhang, Jost B. Jonas

**Affiliations:** 1 Beijing Institute of Ophthalmology, Beijing Tongren Eye Center, Beijing Tongren Hospital, Capital Medical University, Beijing Ophthalmology & Visual Sciences Key Laboratory, Beijing, China; 2 Beijing Tongren Eye Center, Beijing Tongren Hospital, Capital Medical University, Beijing, China; 3 Department of Ophthalmology, Medical Faculty Mannheim of the Ruprecht-Karls-University of Heidelberg, Seegartenklinik Heidelberg, Germany; Medical University Graz, Austria

## Abstract

**Purpose:**

To examine whether *GNAQ* and *GNA11* somatic mutations previously identified in uveal melanomas of Caucasians are associated with uveal melanomas in Chinese patients.

**Methods:**

Uveal melanomas treated by primary enucleation in Chinese patients underwent a mutation analysis of *GNAQ* and *GNA11* with sequencing of exon 5 and exon 4.

**Results:**

The study included 50 patients with uveal melanoma and with a mean age of 47.6±13.0 years. During the follow-up of at least 3 years, 20 (40%) patients developed extraocular metastases. The frequencies of *GNAQ* and *GNA11* somatic mutations in uveal melanoma were 18% (9/50) and 20% (10/50), respectively. The mutations occurred exclusively in codon 209 of exon 5. No mutations were detected in exon 4. Mutations affecting codon 209 in *GNAQ* were c.626A>C(Q209P) (78%) and c.626A>T(Q209L) (22%). Mutations affecting codon 209 in *GNA11* were exclusively c.626A>T(Q209L) (100%). In none of the tumors, mutations of *BRAF* and *NRAS* were detected. *GNAQ*/*11* mutations were marginally (*P* = 0.045) associated with optic disc involvement. In Kaplan-Meier analysis, metastasis-free survival was not significantly (*P* = 0.94) associated with *GNAQ*/*11* mutations.

**Conclusions:**

Mutations of *GNAQ* and *GNA11* can be found in Chinese patients as in Caucasian patients with uveal melanoma, with a higher frequency reported for Caucasian patients.

## Introduction

Uveal melanoma is the most common intraocular malignant tumor in adults, which arises from melanocytes of the choroid, ciliary body, and iris. It accounts for approximately 5% of all melanomas and represents the second most common form of melanoma [1], [2]. Uveal melanoma has a strong tendency to metastasize to the liver and there have been no effective treatments for the metastases. The survival time after the detection of metastases is usually less than 5 to 8 months [3], [4].

An important signaling pathway in the tumor genesis of uveal melanomas is the mitogen-activated protein kinase (MAPK) pathway incorporating RAS, RAF, MEK, and ERK [5], [6]. Previous studies have revealed that cutaneous melanomas showed oncogenic mutations in some components of the MAPKinase cascade, particularly in *BRAF* and *NRAS* [7]–[9]. In contrast, uveal melanomas did not exhibit mutations in *BRAF* and *NRAS* [10]–[11]. Interestingly, the downstream effectors of *BRAF* and *NRAS*, MEK, ERK and ELK, were constitutively activated in uveal melanomas [11]. These mutations remained undetected in uveal melanomas until recently *GNAQ* and *GNA11* mutations in uveal melanoma were identified [12]–[14].

*GNAQ* and *GNA11* encode Gαq, a member of the q class of G-protein alpha-subunits which are involved in mediating signals between G-protein-coupled receptors (GPCRs) and downstream effectors [15], [16]. Recently, oncogenic mutations in the G-protein α-subunit q class were found in about 83% of uveal melanomas with constitutive an activation of downstream MAPK signaling [13]. Glu209 and Arg183 are conserved in the guanosine triphosphate (GTP) binding site of Gα subunits and essential in GTP hydrolysis. The substitutions of glutamine or arginine in *GNAQ* or *GNA11* mutations cause constitutive G-protein activation due to a reduced GTP hydrolysis [17], [18]. Metthew *et al.* reported that R183 mutated oncoproteins were less potent than that of Q209 [19]. *GNAQ* or *GNA11* mutations lead to an upregulating activation of the MAPKinase pathway and appear to be major contributors to the development of uveal melanomas [12], [13].

The landmark studies on the association of uveal melanoma with *GNAQ* and *GNA11* mutations were conducted in Caucasian populations while the status of *GNAQ* and *GNA11* mutations in uveal melanomas of Chinese has not been investigated yet. Considering the genetic variations and their role in tumor genesis in different ethnic groups, we investigated the status of *GNAQ* and *GNA11* mutations in uveal melanoma of Chinese patients to decrypt potential oncogenic differences between Caucasian and Chinese populations. It may promote the understanding of molecular mechanisms and of the pathogenesis of uveal melanomas in patients of different ethnic background.

## Methods

The study included globes which were enucleated for uveal melanomas arising either from the ciliary body or choroid. The study was approved by the medical ethics committee of Beijing Tongren Hospital and was conducted according to the Declaration of Helsinki Principles. All participants gave their written informed consent. The diagnosis of uveal melanoma was substantialized by histological examination of slides stained by hematoxylin and eosin, and by immunohistochemistry showing the melanoma markers of HMB-45, melanin-A and S-100. Clinical and pathologic data including age, gender, largest basal tumor diameter, tumor thickness, histopathologic cell type, ciliary body involvement, optic disc involvement and presence of metastasis were collected. Optic disc involvement was defined as the occurrence of tumor cells at the peripapillary border tissue of Elschnig and Jacoby. For the purpose of our study, the presence of detected metastasis was used as the primary end point. All metastasis-free patients had to have a follow-up of at least 3 years.

The DNA preparation and screening for mutations were performed in several steps. For each tumor, five 5-µm thick, formalin-fixed, paraffin-embedded histological sections were prepared for micro-dissection. Genomic DNA was extracted from the dissected tissues using a QIAamp DNA FFPE Tissue Kit (Qiagen Co., Hilden Germany). To detect hotspot mutations, exon 4 and exon 5 of both *GNAQ* and *GNA11* were amplified by polymerase chain reaction (PCR) in at least two separate preparations of genomic DNA. The technique was described in detail previously [21]. The primer sequences are listed in Table 1. The PCR products were purified with QIAquick (Qiagen Co., Hilden Germany) and sequenced directly.

**Table 1:** Used Primer Sequences.

*GNAQ*exon 5F:	5′-GACTTGGATGATCATCGTCATT-3′
*GNAQ*exon 5R:	5′-AAGAAAGCAAAGAAGTAAGTTCAC-3′
*GNA11*exon 5F:	5′-AGCGTCCTTGCCCGTTCTA-3′
*GNA11*exon 5R:	5′-AGGGCCCACCTCGTTGTC-3′
*GNAQ*exon4F:	5′-TGTCCTTCCCTTTCCGTAGA-3′
*GNAQ*exon4R:	5′-TGGGAAATAGGTTTCATGGACT-3′
*GNA11*exon4F:	5′-GCTGGTTTGGGTGCTGTGT-3′
*GNA11*exon4R:	5′-GGCAAATGAGCCTCTCAGTG-3′

The statistical analysis was performed using a commercially available statistical software (SPSS 21.0, IBM-SPSS, Chicago, USA). The associations between the clinical or pathologic parameters and the mutation types (mutated *GNAQ*, mutated *GNA11*, or neither mutated) were evaluated and the mutational status was categorized into 2 categories (mutated *GNAQ/11* or neither mutated). The Student’s t-test, analysis of variance and Fisher’s exact test were applied. The Kaplan-Meier method and the log-rank test were used for survival analyses. The primary end point for metastasis-free survival was defined as the time to the development of metastatic disease, whereby death due to other causes was treated as censored. *P*-values were based on two-sided tests and, if less than 0.05, were regarded as statistically significant.

## Results

Our study included 50 globes with uveal melanoma treated by primary enucleation. The mean age was 47.6±13.0 years (Table 2). During the follow-up, 10 patients developed extraocular metastases and 10 patients had died from metastases. There were 4 patients as censors with follow-up data before the reference time horizon. All the metastasis-free patients had a follow-up of longer than 3 years. In detail, the metastasis free survival was 94% for 1-year, 90% for 2-years and 87% for 3-years follow-up in the wild type group, 89% for 1-year, 78% for 2-years and 78% for 3-years follow-up in the *GNAQ* mutation group, and 90% for 1-year, 80% for 2-years and 80% for 3-years follow-up in the *GNA11* mutation group.

**Table 2:** Clinical and Pathological Parameters of 50 Patients With Uveal Melanoma.

Clinical-Pathological Parameters	Mean ± Standard deviation (Range) or n (%)
Age (Years)	47.6±13.0 (23–78)
Men/Women	20 (40%)/30 (60%)
Largest Basal Tumor Diameter (mm)	14.7±3.3 (6–20)
Tumor Thickness (mm)	9.5±3.2 (4–20)
Histopathological Cell Type:	
Spindle Cell Type	42 (84%)
Mixed Cell Type	7 (14%)
Epitheloid Cell Type	1 (2%)
Scleral Invasion	32 (64%)
Ciliary Body Involvement	9 (18%)
Optic Disc Involvement	12 (24%)
Metastasis	10 (20%)
Interval from Diagnosis to End Point (Months)	52.3±22.2

To investigate mutations within *GNAQ* and *GNA11*, we amplified mutation hotspot regions (exon 4 and 5) of *GNAQ* and *GNA11* and sequenced the purified PCR products directly. Sequencing of *GNAQ* and *GNA11* was successful in all 50 tumors. Among the 50 samples screened for *GNAQ* and *GNA11* mutations, the overall mutation frequency was 38% (19/50), with 18% (9/50) for *GNAQ* and 20% (10/50)for *GNA11*, respectively. These figures were lower than mutation frequencies reported in Caucasian patients [12]–[14]. In all samples, the *GNAQ* or *GNA11* mutations were mutually exclusive and occurred exclusively at codon 209. Mutations affecting codon 209 in *GNAQ* were c.626A>C(Q209P) (78%) and c.626A>T(Q209L) (22%). These mutations predicted substitution by proline (Q209P) in 78% of samples that were analyzed and by leucine (Q209L) in 22% of the samples. Mutations affecting codon 209 in *GNA11* were exclusively c.626A>T(Q209L) (100%) (Tables 3). These mutations predicted substitution by leucine (Q209L) in all samples analyzed. In addition, all of these tumors had been sequenced for *BRAF* and *NRAS* hotspots, but no mutations were found, which were consistent with previous reports.

**Table 3:** Mutations Found in *GNAQ* and *GNA11* in Chinese Patients with Uveal Melanoma.

Gene	Mutation	n	Total (%)
*GNAQ* exon 5	c.626A>C (Q209P)	7	77.8%
	c.626A>T (Q209L)	2	22.2%
*GNAQ* exon 4	–	0	0
*GNA11* exon 5	c.626A>T (Q209L)	10	100%
*GN11Q* exon 4	–	0	0

Examining correlations between *GNAQ* mutations or *GNA11* mutations and the clinical-pathologic features in univariate analysis revealed a statistically association between the presence of *GNAQ/11* mutations and optic disc involvement (*P* = 0.045) and a statistically weak association between male gender and presence of *GNAQ*/*11* mutations (*P* = 0.04) (Table 4). In a logistic multivariate analysis with *GNAQ/11* mutations as the dependent variable and gender and optic disc involvement as independent variables showed that *GNAQ/11* mutations remained to be significantly (*P* = 0.034; Odds ratio (OR): 4.76; 95% Confidence Interval (CI): 1.12, 20.2) associated with an optic disc involvement while gender was no longer significantly associated (*P* = 0.07; OR: 3.26; 95%CI: 0.92, 11.5).

**Table 4:** Associations of *GNAQ* and *GNA11* Mutations with Clinical-Pathologic Features of Uveal Melanoma in Chinese Patients.

Factors	Total n	*GNAQ* ^mut^, n (%)	*GNA11* ^mut^, N (%)	WT, N (%)	*P*-Value	*GNAQ/11* ^mut^, N (%)	WT, N (%)	*P*-Value
	50 (100%)	9 (18%)	10 (20%)	31 (62%)		19 (38%)	31 (62%)	
Gender								
Men, n (%)	20 (40%)	6 (12%)	5 (10%)	9 (18%)	0.11	11 (22%)	9 (18%)	**0.043**
Women, n (%)	30 (60%)	3 (6%)	5 (10%)	22 (44%)		8 (16%)	22 (44%)	
Age (Years)								
Mean (range)	47.6 (23–78)	43.8 (32–58)	47.6 (25–68)	48.7 (23–78)	0.62	45.8 (25–68)	48.7 (23–78)	0.45
<50, n (%)	27 (54%)	7 (14%)	5 (10%)	15 (30%)	0.34	12 (24%)	15 (30%)	0.31
≥50, n (%)	23 (46%)	2 (4%)	5 (10%)	16 (32%)		7 (14%)	16 (32%)	
Largest Basal Tumor Diameter (mm)								
Mean (Range)	14.7 (6––20)	13.8 (9–20)	15.5 (7–20)	14.8 (6–20)	0.53	14.7 (7–20)	14.8 (6–20)	0.93
<16, n (%)	32 (64%)	7 (14%)	4 (8%)	21 (42%)	0.22	11 (22%)	21 (42%)	0.48
≥16, n (%)	18 (36%)	2 (4%)	6 (12%)	10 (20%)		8 (16%)	10 (20%)	
Tumor Thickness (mm)								
Mean (range)	9.5 (4–20)	9.6 (6–14)	10.7 (5–20)	9.1 (4–16)	0.39	10.2 (5–20)	9.1 (4–16)	0.26
<10, n (%)	26 (52%)	4 (8%)	4 (8%)	18 (36%)	0.61	8 (16%)	18 (36%)	0.27
≥10, n (%)	24 (48%)	5 (10%)	6 (12%)	13 (36%)		11 (22%)	13 (26%)	
Epithelioid Cell Type								
Yes, n (%)	8 (16%)	1 (2%)	3 (6%)	4 (8%)	0.54	4 (8%)	4 (8%)	0.72
No, n (%)	42 (84%)	8 (16%)	7 (14%)	27 (54%)		15 (30%)	27 (54%)	
Scleral Invasion								
Yes, n (%)	32 (64%)	4 (8%)	7 (14%)	21 (42%)	0.41	11 (22%)	21 (42%)	0.48
No, n (%)	18 (36%)	5 (10%)	3 (6%)	10 (20%)		8 (16%)	10 (20%)	
Ciliary Body Involvement								
Yes, n (%)	9 (18%)	0 (0%)	3 (6%)	6 (12%)	0.25	3 (6%)	6 (12%)	1.00
No, n (%)	41 (82%)	9 (18%)	7 (14%)	25 (50%)		16 (32%)	25 (50%)	
Optic Disc Involvement								
Yes, n (%)	12 (24%)	4 (8%)	4 (8%)	4 (8%)	**0.045**	8 (16%)	4 (8%)	**0.045**
No, n (%)	38 (76%)	5 (10%)	6 (12%)	27 (54%)		11 (22%)	27 (54%)	

**Figure 1:**
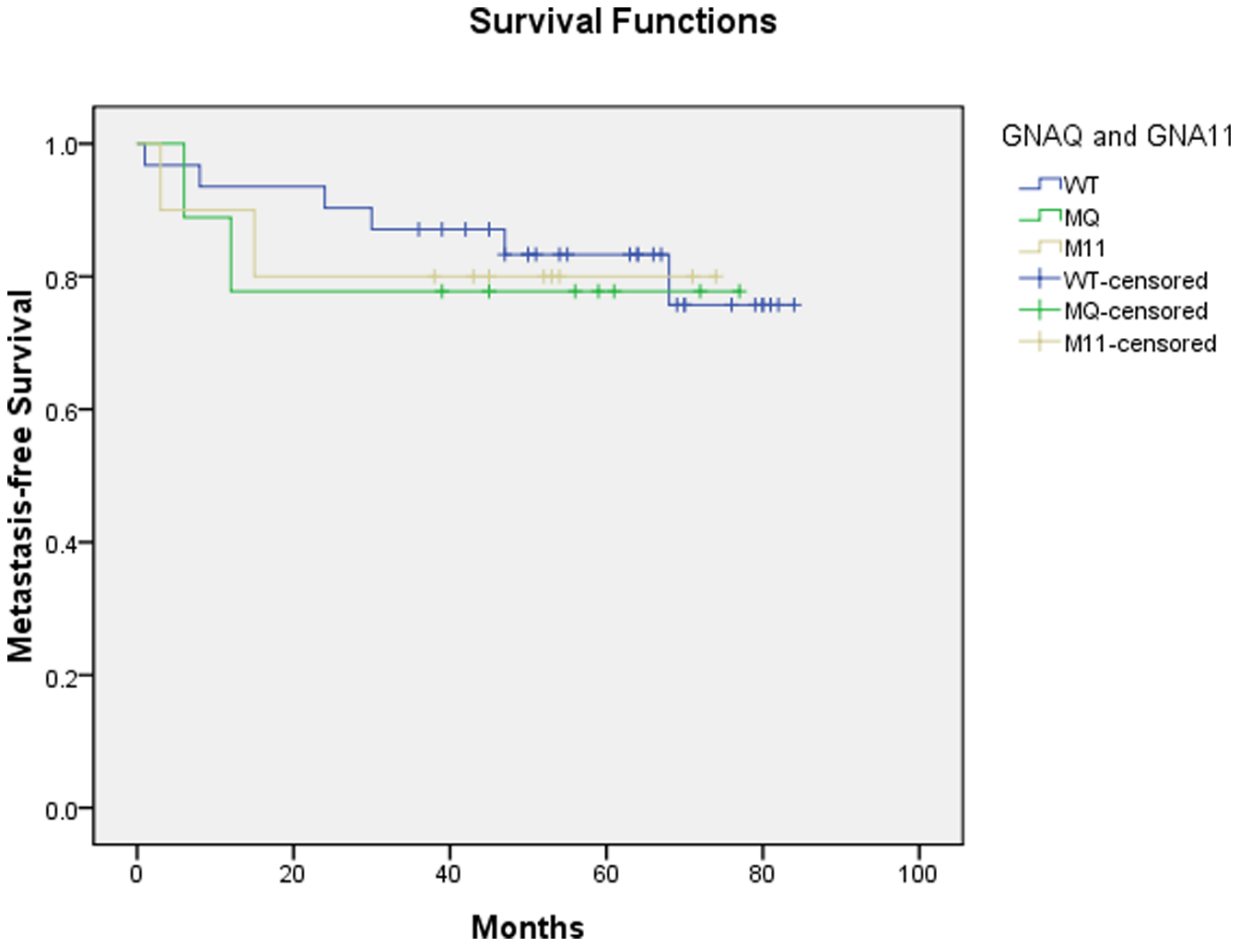
Kaplan-Meier Estimate of Metastasis-Free Survival in Chinese Patients with Uveal Melanomas Harboring either a *GNAQ* or *GNA11* Mutation Compared with Patients with Tumors without These Mutations. WT: Wild type; MQ: *GNAQ* mutation; M11: *GNA11* mutation.

In the Kaplan-Meier analysis, the difference in metastasis-free survival among patients with *GNAQ* or *GNA11* mutations and patients without the mutations was not statistically significant (P = 0.94), indicating that *GNAQ* and *GNA11* mutations were not significantly associated with metastasis (Fig. 1). Due to the relatively small number of patients, a multivariable survival analysis was not performed.

## Discussion

Our hospital-based study showed that mutation frequency of *GNAQ* and *GNA11* in Chinese patients with uveal melanomas was 18% (9/50) for GNAQ and 20% (10/50) for GNA11 with an overall frequency of 38% (19/50). These results on a relatively low percentage of uveal melanomas showing mutations in *GNAQ* and *GNA11* are different from the findings obtained in previous studies on Caucasian patients. Van Raamsdonk and her colleagues found somatic mutations in *GNAQ* or *GNA11* in 83% of the globes with uveal melanomas [13]. The study sample of our investigation was relatively small and was not obtained in a population-based manner to allow a direct comparison of the frequency of *GNAQ* and *GNA11* mutations between different studies. In Caucasian patients with uveal melanomas, the reported *GNAQ* mutation frequency varied between 36% and 53%. Van Raamsdonk and her colleagues detected *GNAQ* mutations in 46% of uveal melanomas [12]. Onken and his colleagues reported that 49% of the uveal melanomas harbored activating mutations in *GNAQ* [14]. In our study, the *GNAQ* mutation frequency was 18%, which was lower than the frequency in studies on Caucasian patients. It has remained unclear however, whether the difference in the reported frequencies of *GNAQ* mutations between the study populations allowed conclusions on inter-ethnic differences.

Interestingly with respect to cutaneous melanomas, the frequency of *BRAF* mutations was 66% in Caucasian patients with cutaneous melanomas while the frequency was only 25.5% in Chinese patients with cutaneous melanomas [9], [20]. Curtin *et al.* reported that mutations of *KIT* gene were detected in 29% of Caucasian patients with cutaneous melanomas while the frequency was only 11% in a Chinese cohort with cutaneous melanomas [21], [22]. These data may suggest that Caucasians and Chinese may differ in the genetic background of cutaneous melanomas. It has remained unclear whether any analogies between cutaneous melanomas and uveal melanomas can be drawn. It has also remained elusive whether the difference in iris and skin pigmentation between Caucasians and Chinese may play a role in the development of uveal melanoma, with fair complexion and light irides being generally considered risk factors for uveal melanoma [23].

Most of the reported *GNAQ* and *GNA11* mutations occurred at codon 209, which was located in the activation domain of this kinase. Van Raamsdonk and her colleagues reported that most of *GNAQ* and *GNA11* mutations occurred at codon 209, and few others occurred at codon 183 [12], [13]. In a similar manner, Onken and his colleagues found that 49% of primary uveal melanomas harbored activating mutations in *GNAQ* at codon 209 [14]. Consistent with previous studies, our data confirmed that *GNAQ* and *GNA11* mutations in exon 5 occurred exclusively in codon 209. Although we did not detect mutations in exon 4, the relatively small number of patients included into our study may not allow concluding that mutations did generally not occur in exon 4 in a study population as ours. Interestingly, mutations in exon 4 appear also in Caucasians in a very low frequency [13].

In previous studies, associations between *GNAQ* and *GNA11* mutations and clinical-pathologic features and prognosis were examined intensively. Van Raamsdonk and her colleagues reported that *GNA11* mutations were more common in locally advanced primary tumors and in melanomas originating from the peripheral choroid or ciliary body [13]. But these associations were not statistically significant. In the study of Onken and colleagues, *GNAQ* mutations were not significantly associated with any clinical or histopathological parameter nor correlated with tumor progression [14]. Onken and coworkers concluded that the *GNAQ* mutation may be an early or initiating event in the tumorgenesis. Koopmans and his colleagues reported that patient survival in uveal melanoma was not correlated with oncogenic mutations in *GNAQ* and *GNA11* [24]. It is in agreement with our study in which *GNAQ/11* mutations were not significantly associated with age, largest tumor basis diameter, tumor thickness, tumor cell type, scleral invasion or ciliary body involvement, with these parameters being associated with a poor prognosis of uveal melanomas. Consequently, we also did not find statistically significant associations of *GNAQ/11* mutations with metastasis.

Interestingly, *GNAQ/11* mutations were associated with uveal melanomas involving optic disc in our study. Future studies may address whether this finding could be due to site-specificity. Specific windows for *GNAQ* signaling in terms of location, cell type and developmental time were previously reported, such as that *GNAQ* mutations induced melanocytic proliferations spared epithelial structures [16]. Our study did not allow addressing whether the association between *GNAQ/11* mutations and uveal melanomas involving optic disc would be a parallel to the previous observation of two choroidal melanocytomas harboring mutations in *GNAQ* with one of them transforming into uveal melanoma [25].

Potential limitations of our study should be mentioned. First, it was a hospital-based study with the inherent risk of a referral bias. Second, the study sample was relatively small, so that the power of the statistical analysis was limited. Strength of the study was that it was the first study to investigate the frequency of GNAQ/11 mutations in uveal melanomas of Chinese patients.

In conclusion, mutations of *GNAQ* and *GNA11* can be found in Chinese patients as in Caucasian patients with uveal melanoma, with the reported frequency being higher in Caucasian patients. Future studies may address whether as suggested by our study, the frequency of these mutations is indeed lower in Chinese than in Caucasians.
